# Peritoneal Dialysis in Young Adults: A Mixed-Methods Study

**DOI:** 10.1016/j.xkme.2025.100983

**Published:** 2025-02-15

**Authors:** Hannah C. Lyons, Lucy E. Selman, Yoav Ben-Shlomo, Fergus J. Caskey, Carol D. Inward, Alexander Hamilton

**Affiliations:** 1 Population Health Sciences, Bristol Medical School, University of Bristol, Bristol

**Keywords:** Peritoneal dialysis, Young adults, Chronic kidney disease, Haemodialysis

## Abstract

**Background:**

Peritoneal dialysis (PD) preserves kidney function and offers flexibility; however, few young adults have it compared with hemodialysis (HD). This study aimed to understand factors influencing the change from PD to HD.

**Study Design:**

This was a sequential explanatory mixed-methods study.

**Setting & Participants:**

Quantitative data were collected from 470 participants (50% male participants, 85% White, mean age: 16 years) who received dialysis between 1987 and 2015. Cox proportional hazards analysis was used to examine psychosocial factors associated with transitions from PD to HD. Qualitative data were gathered from 13 young adults (aged 14-29 years) who received dialysis between 2013 and 2015, with retrospective interviews conducted in 2020.

**Results:**

25% of participants experienced multiple episodes of PD. Survival rates for PD at 1 and 5 years were 71% and 37%, respectively. Risk factors for transitioning to HD included young adulthood (age: 15-30 years), with higher transition risks in older age groups (age: 15-19 years, HR: 2.41; age: 20-24 years, HR: 3.39; age: 25-30 years, HR: 3.14; *P* < 0.005). Other factors included primary kidney disease type (systemic diseases vs tubulointerstitial diseases). Leading causes for transition were infection (50%), noncompliance (21%), and mechanical issues (18%). Qualitative analysis revealed the key themes around communicating treatment options, life impact, and support structures. Resilience was an additional theme among those who continued PD.

**Limitations:**

The study was based on cross-sectional psychosocial data, lacked detailed parental involvement, and may have suffered recall bias.

**Conclusions:**

Young adults are at higher risk of transitioning to HD owing to both transplant failure and complications with PD. Challenges of PD have been underestimated, and there is a need to educate young adults well on all dialysis options. Additional support including mental health support, peer support, and support during life changes, such as moving out of their family home, is recommended.

## Plain-Language Summary

This study aimed to understand why young adults are more likely to switch from peritoneal dialysis (PD) to hemodialysis. Although PD preserves kidney function and offers flexibility, few young adults choose it. We conducted a mixed-methods study with 470 patients aged 0-30 years and interviews with 13 individuals who were receiving dialysis. Our findings showed that young adults (aged 15-30 years) were at higher risk of transitioning to hemodialysis, mainly owing to infections, noncompliance, and mechanical issues. Challenges of PD have been underestimated, and there is a need to educate young adults well on all dialysis options. Additional support including mental health support, peer support, and support during life changes, such as moving out of their family home, is recommended.

Chronic kidney disease (CKD) affects over 17,000 young people aged 16-34 years in England. Although most individuals with advanced CKD eventually receive a kidney transplant, 24% of young adults receive hemodialysis (HD), and only 6% receive peritoneal dialysis (PD).^1^ PD offers several potential benefits for young adults, including the preservation of residual kidney function, improved vascular access, greater autonomy through flexible treatment schedules, and the ability to travel.^2^ Despite these advantages, PD is less commonly used among young adults, and the reasons for this are not fully understood.

The question of whether young adults are at higher risk of transitioning from PD to HD (PD-to-HD transition) compared with other age groups remains underexplored, as this population has often been underrepresented in previous studies.^3^^,^^4^ There is little known about whether young adults are at increased risk of numerous factors linked to PD-to-HD transition, including inadequate dialysis, peritonitis, and late nephrology referral.^5^, ^6^, ^7^, ^8^, ^9^, ^10^

However, there are some factors linked to PD-to-HD transition that young adults are known to be at increased risk of. These include unemployment, refraining from relationships, body image concerns, and medication compliance.^11^, ^12^, ^13^, ^14^, ^15^

There is a need for further evidence on the factors associated with PD-to-HD transition in young adults, as well as a deeper understanding of their experiences on receiving PD. Such insights can help clinicians better inform patients about potential risks and treatment options, enabling shared decision making about whether PD remains a viable option or whether HD may better meet their needs.

This study aims to explore associations with PD-to-HD transition using retrospective data and to further illuminate these findings through qualitative interviews that examine the lived experiences of young adults who have received PD.

## Methods

We used an explanatory sequential mixed-methods study design. This comprised a quantitative analysis in a retrospective cohort, followed by semistructured qualitative telephone interviews to explore the findings.

### Retrospective Cohort

We used data collected by the Surveying People Experiencing Young Adult Kidney Failure study.^1^ This was a cross-sectional observational survey study of 16- to 30-year-old participants receiving kidney replacement therapy from 1987-2015 across the United Kingdom, which included 74 sites and linked to the UK Renal Registry, which includes data on treatment changes.^1^ The study excluded those psychologically unfit to participate or unable to complete the questionnaire with assistance.

Quantitative analysis involved constructing a theoretical framework (Item S1) based on literature review to explore psychosocial associations with PD-to-HD transition, censoring for transplantation. Because timeline data were retrospective and survey data cross-sectional, only variables considered consistent over time were included.

Individual timeline data (1987-2015) identified PD exposures and outcomes such as remaining on PD, switching to HD, transplantation, loss to follow-up, or death. Because multiple treatment failures per patient could occur, a conditional risk set for multiple failure data was created with age group as time varying.^16^^,^^17^ Statistical analyses included Kaplan-Meier survival curves and log-rank tests for univariate associations. Using backward elimination, associated variables (*P* < 0.1) were combined in a multivariable Cox proportional hazards model.^18^ Explanatory variables were removed from the ﬁnal model if there was no statistical association when coadjusted, having checked that this did not affect the regression coefﬁcients of the remaining variables.^18^ The model used clustering at the participant level and stratification based on failure number.^18^ All statistical analyses were performed using Stata 15.1 (StataCorp).

### Qualitative Interviews

An interview guide (Item S2) informed by the literature review and regression analysis findings was developed.

Study invitation letters were sent to 87 traced surviving patients across the United Kingdom who had received PD during 2013-2015 and were aged between 15 and 30 years at the time they had received PD. Twenty consented to participate, and from these, 13 participants were purposively sampled based on their demographics and if they had experienced PD-to-HD transition to increase representativeness. Interviews were done in 2020 by an independent researcher with training on how to conduct qualitative interviews and use the interview guide (HCL). Interviews focused on pre-PD experiences, treatment encounters, and reasons for PD-to-HD transition. Most participants (n = 12) had undergone kidney transplant, whereas 1 had recovered kidney function. Hence, none were still receiving PD when interviewed.

Thirteen respondents were interviewed; 11 were White and 7 were male participants. Interviews lasted 40-120 minutes. Participants’ mean age was 23 years (range: 14-29 years) when they received PD. Seven participants continued to receive PD, 5 changed from PD to HD once, and 1 changed from PD to HD twice. Primary kidney disease included glomerular diseases (n = 7), tubulointerstitial disease (n = 4), systemic diseases (n = 1), and miscellaneous kidney disorders (n = 1).

The interviews, conducted from April 2020 to December 2021, aimed to retrospectively explore young adults’ experiences with PD. Quantitative findings were used to guide the development of the interview guide. Telephone interviews were chosen owing to the coronavirus disease 2019 pandemic. A distress protocol was available but not required.

Thematic analysis, employing deductive and inductive approaches, aimed to provide deeper insights into the factors influencing PD-to-HD transition, as well as draw comparisons with HD (which 6 participants had also experienced).^19^ NVivo 12 (QSR International) was used to manage data analysis, with coding initially done independently by 3 researchers (HCL, AH, and LES) before a consensus on a refined framework was achieved.^19^, ^20^, ^21^

Transcripts were categorized based on PD-to-HD transition status to identify potential data patterns.^22^ Reporting adhered to the Consolidated Criteria for Reporting Qualitative Research guidelines.^23^ Qualitative data were analyzed further to gain further insights into the quantitative data, from the most common causes of PD-to-HD transition to why certain age groups are more at risk of PD-to-HD transition.

The Surveying People Experiencing Young Adult Kidney Failure study was granted ethical approval by the Health Research Authority National Research Ethics Service Committee South West-Cornwall & Plymouth (15/SW/0101). Additional ethical approval for the qualitative interviews was granted by the University of Bristol Faculty of Health Sciences Research Ethics Committee (96370).

## Results

In the retrospective cohort study, among the 976 Surveying People Experiencing Young Adult Kidney Failure study participants, timeline data were available for 911 participants. Of these, 470 had received PD and 135 had multiple PD exposures. The cohort’s mean age was 16 ± 8 years. Demographic and psychosocial characteristics are shown in Table 1.^24^^,^^25^

**Table 1:** Demographics of the Study Population Used in Quantitative Research at the Time of Survey

Variable	Total n	n	%
Male sex	470	236	50.21
Number of PD entries in treatment timeline	470		
1		335	71
2		99	21
3		26	6
4 or more		10	2
PD failure	605^a^	208	34
Age group (y) at PD failure event	605^a^		
0-4		69	11
5-9		61	10
10-14		102	17
15-19		155	26
20-24		146	24
25-30		72	12
Ethnic group	470		
White		399	85
Asian		47	10
Black		12	3
Mixed/other		12	3
Late nephrology referral (>90 d)	247	91	37
Modality change (any change among PD, HD, and transplant) >90 d	321		
0		36	11
1		142	44
2+		143	45
Religion	245		
No religion		159	65
Christian		59	24
Muslim		19	8
Any other religion		8	3
Primary renal disease group^b^	463		
Glomerular disease		148	32
Systemic diseases affecting the kidney		36	8
Familial/hereditary nephropathies		59	13
Tubulointerstitial disease		137	30
Miscellaneous kidney disorders		83	18
Index of multiple deprivation quintile^c^	321		
1 (most deprived)		54	17
2		81	25
3		50	16
4		74	23
5 (least deprived)		62	19
Age on finishing secondary school education (y)	296		
Not yet finished (still attending secondary school)		55	19
Never went to school		2	1
15 or under		6	2
16-17		66	21
18 or above		167	56
Higher education or degree	287	107	37
Able to drive	294	170	58
Has children	296	41	14
Independence with Activities of Daily Living scale score^d^	279		
>26 (not fully independent)		111	39
26 (fully independent)		168	60
Patient Activation Measure-13 group	244		
Level 1 – least activated		60	25
Level 2		46	19
Level 3		88	36
Level 4 – most activated		50	20
Patient Satisfaction Questionnaire-18 subscale^e^	253		
Low		26	10
Medium		64	25
High		163	64
Body Image Score^f^	264		
Median (interquartile range)		10.5	4, 19.5
Social Impact Score^g^	237		
Median (interquartile range)		42	29, 54
Multidimensional Scale of Perceived Social Support^h^	252		
Median (interquartile range)		68	56, 76

Abbreviations: HD, hemodialysis; IMD, index of multiple deprivation; PD, peritoneal dialysis.

a Some patients had multiple episodes of PD (eg, at 4 y and 16 y).

b According to the 2012 European Renal Association–European Dialysis and Transplant Association coding system.^24^

c Derived United Kingdom–wide IMD quintiles using postcodes.^25^

d This is a score attributed to whether individuals can complete everyday activities such as getting dressed independently or if they need assistance.

e Refers to their satisfaction with the health care service they received (including aspects such as communication, time spent with the doctor, and accessibility).

f Possible range 0-30; higher scores indicate a more negative body image change or dissatisfaction.

g Possible range 21-88; higher scores indicate greater stigma.

h Possible range 16-84; higher scores indicate greater social support.

**Figure 1:**
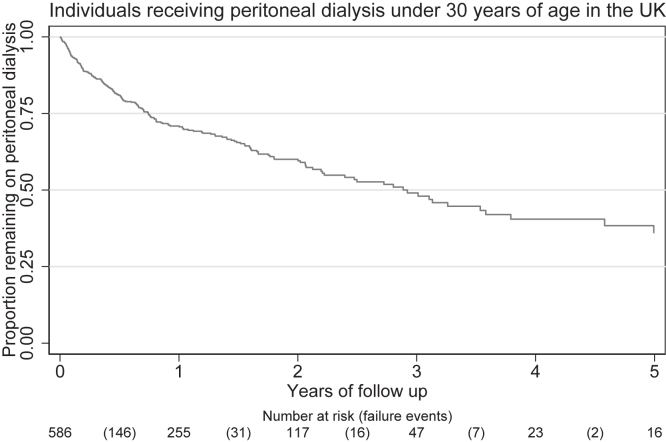
Kaplan-Meier peritoneal dialysis treatment survival curve.

Of the 609 PD episodes spanning a 757-year risk period, 209 resulted in PD-to-HD transition. Notably, 64% (n = 301) continued receiving PD, whereas 29% (n = 137) had 1 PD-to-HD transition and 7% (n = 32) experienced multiple PD-to-HD transitions. The majority of those who continued to receive PD went on to have a kidney transplant, whereas a few recovered. The median time to PD-to-HD transition was 0.9 years, with a 12-month survival rate of 71% (95% confidence interval, 67-75), declining to 37% (95% confidence interval, 28-46) at 5 years, as shown in Figure 1.

The Cox proportional hazards model indicated increased PD-to-HD transition risk for those aged 15-30 years (compared with those aged 10-14 years) and for systemic diseases over structural diseases. Table 2 shows the hazard ratios of variables from the Cox proportional hazards model. Among systemic diseases (n = 43), renovascular diseases had the highest rate of PD-to-HD transition (64%), compared with other conditions including diabetes (50%), hemolytic uremic syndrome (42%), and kidney vein thrombosis (29%); *P* = 0.5 (Fisher exact test). In cases with available data (n = 101), infection (50%), adherence issues (21%), and mechanical problems (18%) were the primary reasons for PD-to-HD transition.

**Table 2:** Cox Proportional Hazards Model for Peritoneal Dialysis Failure Showing Multivariate Cox Regression Analysis

Variable	Hazard Ratio	95% Confidence Intervals	*P* Value
Sex			
Male	0.92	0.69-1.24	0.6
Age group (y)			
0-4	1.15	0.57-2.33	0.7
5-9	1.17	0.60-2.27	0.6
10-14	1.00		
15-19	2.41	1.35-4.28	0.003
20-24	3.39	1.97-5.83	<0.0001
25-30	3.14	1.65-6.00	0.001
Primary renal disease group			
Tubulointerstitial diseases	1.00		
Glomerular diseases	1.36	0.94-1.95	0.1
Systemic diseases affecting the kidney	1.97	1.12-3.46	0.02
Familial/hereditary nephropathies	1.19	0.74-1.91	0.5
Miscellaneous kidney disorders	0.84	0.51-1.39	0.5

### Qualitative Findings

**Figure 2:**
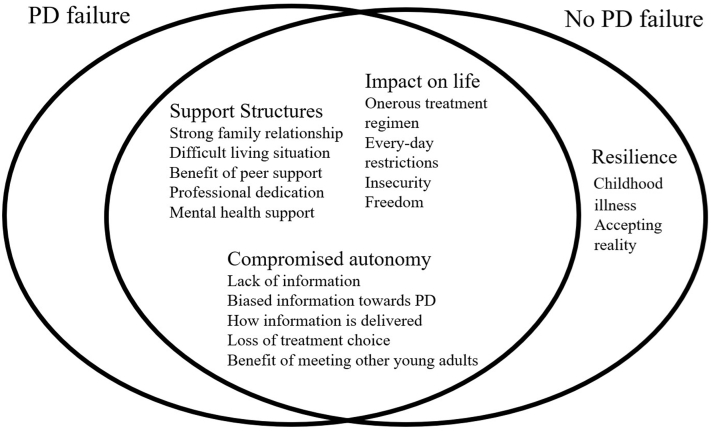
A thematic schema of young adults’ experiences of peritoneal dialysis. PD, peritoneal dialysis.

We identified the following 4 main themes: (1) communicating treatment options; (2) impact on daily life; (3) support structures; and (4) resilience. These themes and the subthemes are illustrated in Figure 2. The narrative cross-references to exemplifying data extracts are provided in Table 3.

**Table 3:** Themes, Subthemes, and Illustrative Quotations

Theme	Subtheme	No.	Exemplifying Quote (Q) With Interviewee Number, Sex, and Age (y)
1. Communicating treatment options	1.1 Lack of information	Q1	“About the hemo, yeah because obviously I didn’t get any information about it really.” – P1 (M, 22^a^)
		Q2	“When you drain the last of the bag out for the next one to go in... I think everyone gets it ... a stingy feeling where its trying to just get the last bits out... It definitely wasn’t mentioned ... and when we did the training perhaps on ourselves I first got that and then it was like oh yeah yeah, that happens sometimes.” – P2 (M, 23)
		Q3	“I was never told any information about the kind of changes to my body that I would experience which was a big issue.” – P3 (F, 19^b^)
	1.2 Biased information toward PD	Q4	“I did feel the emphasis was heavily on peritoneal.” – P4 (M, 23)
		Q5	“Oh, it’s really easy, you know, you won’t have any issues, you probably won’t have to deal with half of the things that we’ll talk about.” – P5 (F, 20)
	1.3 How information was delivered	Q6	“Yeah they were A4 pieces of paper just top to bottom full of text… I did have a brief look… I think leaflets were quite heavy with text and they had quite a few pages… I mean a booklet with lighter text or spread across many pages rather than condensed on a few pages.” – P6 (M, 25)
		Q7	“it just put me off the hemodialysis because obviously it was in the hospital. So the people that were there were the people that need to be in hospital at the same time so they weren’t the healthiest of hemodialysis patients … if you were to go to a satellite unit the hemodialysis patients there are a complete opposite which I didn’t find out until later really.” – P1 (M, 22)
		Q8	“I felt pretty confident with it and then they gave us like a list of step by step instructions to follow and obviously sort of follow those without … follow the procedure, you can’t go too wrong.” – P2 (M, 23)
	1.4 Loss of treatment choice	Q9	“I wasn't given the option so I kind of, yeah, didn’t really think of asking.” – P7 (M, 26)
		Q10	“Why are you putting these additional tubes in me which we told you wouldn’t, even it were to work it wouldn’t be the best option, it’s not what we wanted, it was almost forced upon us… Everything we were trying to say was falling upon deaf ears and it was almost as if these are your options but we’re going to steer you towards the one set path of having dialysis at home, as much as that didn’t work or suit what I felt was my personal situation.” – P6 (M, 25)
	1.5 Benefit of meeting other young adults	Q11	“There was a couple of teenagers … who were on home dialysis as well as HD … that’s how me and my parents decided which type of dialysis I was going to do. Because I had the opportunity to talk to young like kind of same age people.” – P9 (F, 14)
		Q12	“Explained a little bit more in depth really perhaps by somebody who had a tube but didn’t sort of tiptoe around the idea and just sort of said the good things and the bad things.” – P1 (M, 22)
		Q13	“If I’d spoken to someone who’d been through it before it actually got into that stage that probably would have been quite handy … information on best ways of sort of doing things, sort of just probably reassurance to know that it’s not going to sort of impact your life.” – P9 (M, 28)
2. Impact on daily life	2.1 Onerous treatment regimen	Q14	“But to be able to get up in time to go to work I'd have to, sort of, make sure that I was in bed on the dialysis by really 9 o'clock so that it would finished at 7 and then I'd be able to get up and get ready for work?… I couldn't really go out with my friends of an evening. Like say, like, they were all going out for tea, obviously I'd be able to go for maybe a little bit but I was still, sort of, really restricted on what I could eat and drink because of the dialysis. And then I'd have to leave early so that I could get back and get on the machine and stuff like that. So it, like, really impacted my social life. And then, obviously like, just being hooked up to a machine all night it's not very nice… I was quite angry that I have to, sort of, just be on that machine every day and you just didn't really get a break from it.” – P11 (F, 23)
		Q15	“Having that midday exchange and then having to like you know connect myself again, it’s just overwhelming… Like not having someone who was like checking the stock, then coming home and also having to deal with that, it was just too much.” – P10 (F, 20^c^)
		Q16	“Just because you don't have to do it 4 times a day is probably the biggest thing… Once you leave that satellite unit, you don’t have to think about it for a couple days.” – P1 (M, 22)
	2.2 Everyday restrictions	Q17	“And the drawbacks, the amount of washing your hands, all the boxes … the diet is quite hard, sticking to the diet and there was so many restrictions it was hard.” – P7 (M, 26)
		Q18	“And then when you’re spending so much time literally like tethered to the machine it can feel a little bit difficult at times, a little bit depressing when, I don’t know, 7, 8 pm you’re sort of in bed already and everybody else is still out and about and doing things.” – P4 (M, 23)
		Q19	“I’m quite an active person, I play sports couple of times a week and then I sort of stopped doing that in case of sort of, you know, I was getting hit on it or anything like that, not risk anything.” – P6 (M, 25)
		Q20	“My friends sometimes at football I’d be doing, you know, I was struggling to run and I’d be a bit lazy and they’d get on me a bit like come on, stop being so lazy and laugh, you know, they could forget as much as … people can forget that you aren’t actually normal but I did like to see myself as normal.” – P7 (M, 26)
	2.3 Insecurity	Q21	“Probably the sexual side of it to be honest. Obviously, you get sexual with someone, you’re there, they try to take your top off, you might have to pull it down or … you know what it’s like. Sort of what if it scares them off and things, yeah so you don’t tend to do it really … sort of be grossed with it really.” – P1 (M, 22)
		Q22	“I did develop an eating disorder. No-one weighs 40 kilos without it.” – P5 (F, 20)
		Q23	“I can’t sit by myself and just enjoy being myself because it’s almost like just flashbacks of being forced to be by myself for such a long period of time.” – P3 (F, 19)
	2.4 Freedom	Q24	“I think certainly the main benefits are that you’re on it (PD) through the night and so you can live a relatively normal life from home and you’re doing the dialysis at home, so you’ve got quite a lot of freedom there rather than having to go to a hospital.” – P7 (M, 26)
		Q25	“It (PD) worked much better that I had the overnight, but I had the option to do the day ones… It was much more freedom, like I could do a lot of stuff, like I didn’t have to sort of like worry about being back for a certain time or having to change days that I would dialyse.” – P2 (M, 23)
		Q26	“It gave me the flexibility in the day to be free and work still, so I was still able to work full-time, that’s why I picked it rather than the hemodialysis at the hospital ‘cos it would have meant me not being able to keep my job. So it enabled me to keep my job and then sort of have my own financial responsibilities and therefore gives me more freedom ‘cos I had my job and my money and my independence and stuff.” – P4 (M, 23)
3. Support structures	3.1 Strong family relationship	Q27	“I was quite fortunate to have sort of family and things that were very supportive at the time and would help… I mean some days you can still feel quite unwell. You can still feel sick with the toxins building up in your body, … just a bit tired, just wanna not do it.” – P1 (M, 22)
		Q28	“I remember like sitting in my bedroom with my sister and my Mum and we all like in the doorway, me and Mum finished cleaning everything, like disinfecting the machine, disinfecting everything and washing my hands.” – P10 (F, 14^c^)
		Q29	“I think I would have struggled a lot more now ‘cos I live with my fiancé now... I don’t think I would have been able to cope as good ‘cos my mum was like second nurse kind of thing so, you know, looking after me and kind of doing all my food and meals and stuff like that and encouraging me to eat and doing everything for me in that respect and stuff.” – P12 (F, 21)
	3.2 Difficult living situations	Q30	“I had to explain to like 4 messy um roommates why they had to keep the house clean, and especially like the bathroom area… There were some disagreements and fights about it… Having arguments like oh my god there’s huge boxes everywhere… I think that the biggest advice someone could have given me if you’re … to live on your own… But if you’re living away from home, the reason I got peritonitis was because I was living with other people, keeping on top of hygiene, keeping the peace in the house with all the deliveries was a task in itself… If somehow, I managed to like move in somewhere on its own, obviously it has its own financial impact, and for me that wasn’t really like an option like financially.” – P5 (F, 20)
		Q31	“It was literally always looming over me… I couldn’t escape it because sitting in my room there was stuff everywhere.” – P3 (F, 19)
	3.3 Benefit of peer support	Q32	“I think that was the thing that was missing was that emotional support. I was never offered that opportunity to talk to anyone, I was never offered the opportunity to have someone that understood, even if it was just meeting other people that were going through it or other young people or something that would have allowed me to have someone to talk to.” – P3 (F, 19)
		Q33	“I remember I was doing my dialysis (hemodialysis) and we ended up having a laugh and joke about all of that stuff ... it does help to have the people with you and you’ve built up rapport and a bit of chat and stuff like that, ‘cos obviously you’ve got a lot in common at that particular time.” – P6 (M, 25)
		Q34	“It probably just wouldn’t have interested me to go somewhere just to like purposely talk about it when sort of like every time I normally see someone that’s all I ever seem to talk about anyway. I’ve had enough of kind of talking about it, if you know what I mean… Yeah, if I’d had sort of someone my age to speak to at the start of it to kind of reassure you and sort of go through bits like that, I think that probably would have been quite handy.” – P13 (M, 29)
	3.4 Professional dedication (ability to travel, transport provided to satellite units)	Q35	“I think people who surrounded me handled me very gently and calmly and I think that really helped… I think it was just kind of like treated me like an adult at that age, of saying like ok it’s tough, we’ll pause it, let you breathe for a bit, leave you for 15 minutes and then come back to you. Kind of like reassurance but also like giving me space.” – P10 (F, 14^c^)
		Q36	“Travels also possible coz I mean I went on holiday with it to Spain… And peritoneal, the level of support if you’re hoping to travel was second-to-none really.” – P1 (M, 22)
		Q37	“I’m going to get told well you’re being a hysterical girl again… I was telling them that the dialysis wasn’t working properly, in the end I actually stopped doing the dialysis altogether, I spent about 6 months not doing it because they weren’t listening.” – P5 (F, 20)
	3.5 Mental health support	Q38	“I think issues would have been picked up on a lot, lot sooner had there been someone I could talk to … it’s that kind of situation of I can’t believe for something that is so isolating or potentially isolating and such a big thing to take on, I find it so difficult to believe that there isn’t more mental health support.” – P3 (F, 19)
		Q39	“At the time then I didn’t realise I was feeling really low in myself, like psychologically.” – P10 (F, 14^c^)
		Q40	“It got progressively worse over about 3 y I think to be honest until I actually had started seeking the help. But once I did, I just sort of generally mentioned once when I was in hospital and all of a sudden, I had this new level of support which I didn’t know existed really. Actually, I managed to see a therapist every week then which was very helpful.” – P1 (M, 22)
4. Resilience	4.1 Childhood illness	Q41	“I first had kidney failure when I was 5 so had a kidney transplant first of all when I was 9 y old so for those 4 y between I had, I was a child obviously, you had all the dialysis in the hospital, I had it at home and it was kind of and then I was always on check-ups after that and we always knew that at some point down the line it would kind of fail and I would have to go through the process again. So, it wasn't like a huge surprise we knew it was kind of coming… I was more aware obviously, having had it as a child.” – P4 (M, 23)
		Q42	“So, I think because from like age 8 I already knew kind of like knew what like permanently ill, or having chronic disease was, I didn’t find it like extremely overwhelming. At that point like already been really sick, in and out of hospital. And it almost like a certain point, a year of being dialysis at home was a relief, a relief I wouldn’t have to spend a week in a hospital without like seeing my Mum every night or like my sister.” – P10 (F, 14^c^)
	4.2 Accepting reality	Q43	“I think it was just adjustment. It was like, I don’t know, you know when in the morning you have your own personal routine of making coffee, then you go and... It was like the same kind of thing that you’re like, once you’ve done it enough times it just becomes almost like natural and it would seem very weird if you didn’t do it that way.” – P10 (F, 14^c^)
		Q44	“Well I just didn’t want... I wanted to carry on living my life on dialysis rather than dialysis take over my life. So, I was adamant… I didn’t want it to stop me doing things. But like I said it was getting hard at the end, especially a young age, it’s hard for young people.” – P7 (M, 26)
		Q45	“18 months was enough … I’d had enough of being on dialysis and just wanted to crack on with my life.” – P2 (M, 23)

Abbreviation: PD, peritoneal dialysis.

a F, 19 indicates this participant was a female patient aged 19 y.

b M, 22 indicates this participant was a male patient aged 22 y.

c This participant had 2 experiences of PD, aged 14 and 20 y. Her experiences were explored separately in the interview.

#### Communicating Treatment Options

The following 5 subthemes were identified relating to communicating treatment options: (1) lack of information; (2) biased information toward PD; (3) how information was delivered; (4) loss of treatment choice; and (5) benefit of meeting other young adults.

### Lack of Information

Patients expressed dissatisfaction with the information provided about dialysis options (Q1). Some patients (n = 7) were not informed about HD, whereas others (n = 5) lacked awareness of the discomfort and bodily changes associated with PD (Q2 and Q3).

### Biased Information Toward PD

Most participants (n = 12) felt health care professionals exhibited bias toward PD, assuming it as the default choice for young patients (Q4). Some participants (n = 5) criticized the unrealistic portrayal of PD, highlighting misconceptions about its ease and lack of issues (Q5).

### How Information Is Delivered

Information delivery methods were criticized (n = 10) for being text dense and insufficiently informative, with few opportunities for practical exposure (such as visiting a satellite HD unit) (Q6 and Q7). However, clear instructions on PD procedures were praised (n = 9), which enhanced their confidence in managing the treatment (Q8).

### Loss of Treatment Choice

Most participants (n = 10) felt deprived of treatment choice, expressing they were steered toward PD and wished they had had more options (Q9 and Q10).

### Benefit of Meeting Other Young Adults

Interactions with other young adults receiving dialysis emerged as beneficial, aiding in decision-making processes and providing a greater understanding of the treatment’s realities (Q11). Others expressed a desire to meet others with personal experience of PD to gain insights into their experience and practical tips (Q12 and Q13).

#### Impact on Daily Life

The following 4 subthemes were identified relating to the impact of PD treatment on daily life: (1) onerous treatment regimen; (2) everyday restrictions; (3) insecurity and low self-esteem; and (4) freedom.

### Onerous Treatment Regimen

Most participants (n = 10) found the extensive time spent cleaning, preparing, and performing PD daily overwhelming. One also expressed that assisted PD was too onerous owing to having to arrive home early every evening, to set it up, and to wake up early before work (Q14). One found the high frequency of PD exchanges too burdensome (Q15). Some participants (n = 4) preferred HD owing to its less-frequent schedule, emphasizing the importance of having days off dialysis (Q16).

### Everyday Restrictions

Everyday restrictions were reported by most participants (n = 10), ranging from dietary limitations to social constraints in which they had to come home early to perform PD (Q17 and Q18). Four male participants refrained from sports to avoid damaging their PD tube, which had a detrimental psychological impact, whereas some (n = 2) found sports helpful for coping (Q19 and Q20).

### Insecurity and Low Self-Esteem

Some refrained from sexual activity or seeking relationships, with both male and female participants fearing rejection owing to their PD catheter (n = 7) (Q21). Female participants expressed concerns about their appearance (such as being bloated), with one developing an eating disorder (Q22). Performing PD alone at home heightened the feelings of anxiety and insecurity (n = 4) (Q23).

### Freedom

However, some felt that PD provided them with freedom (n = 4). They appreciated the ability to performing PD overnight or during the day, which allowed them to stay employed (Q24, Q25, and Q26).

#### Support Structures

The following 5 subthemes were identified relating to the support structures participants relied on: (1) strong family relationships; (2) difficult living situations; (3) benefit of peer support; (4) professional dedication (ability to travel and transport provided to satellite units); and (5) mental health support.

### Strong Family Relationships

Most participants (n = 11) attributed their PD success to having supportive families actively involved in their treatment, such as those involved in cleaning and disinfecting the machine, especially on days when they felt too unwell to manage it alone (Q27 and Q28). Participants expressed how they transitioned from PD to HD when they moved out of their family homes (Q29).

### Difficult Living Situations

Several participants faced challenges when living independently, especially in shared accommodation (n = 7). Issues such as limited space for storing equipment and maintaining hygiene standards in student houses posed problems (Q30). Patients who experienced PD failure struggled to find suitable storage solutions, exacerbating their psychological burden (Q31).

### Benefit of Peer Support

Peer support emerged as beneficial for coping. Some participants expressed a desire to connect with others of their age undergoing PD, believing that shared experiences could alleviate the challenges they faced (n = 6) (Q32). Those who engaged with peers described finding comfort in shared understanding (Q33). However, one person was reluctant to talk more about PD (Q34).

### Professional Dedication (Ability to Travel and Transport Provided to Satellite Units)

Health care professionals played a crucial role in patients’ PD experiences, with most participants (n = 8) praising their dedication and support (Q35). Patients appreciated professionals who balanced being caring and patient with treating them as adults (Q35). Moreover, they valued the practical training and 24-hour support they received. All who traveled abroad (n = 4) praised the great level of support for this (Q36). However, participants who experienced strained relationships with health care providers, characterized by dismissiveness or lack of empathy, were more likely to develop PD failure (Q37).

### Mental Health Support

Mental health support emerged as a pressing need among PD patients, with the majority (n = 9) reporting mental health issues following treatment. Many were initially unaware of their declining mental health, underscoring the importance of timely recognition and intervention (Q38 and Q39). When mental health support was sought, participants (n = 4) expressed that they were impressed by it (Q40). The need to raise awareness that this support is available was emphasized.

#### Resilience

This was a specific theme for those who had success with PD. The following 2 subthemes were identified relating to resilience: (1) childhood illness and (2) accepting reality.

### Childhood Illness

Three out of 7 participants found that their childhood experiences with kidney disease prepared them for dialysis, with one expressing relief that dialysis meant avoiding prolonged hospital stays (Q41 and Q42).

### Accepting Reality

Five out of 7 participants expressed that accepting PD as part of their daily routine proved beneficial and consequently fostered determination to keep socializing (Q43). However, resilience sometimes faltered over time, with some feeling fatigued by dialysis (Q44).

## Discussion

This study highlights young adulthood as a particularly vulnerable period for individuals undergoing kidney replacement therapy, offering valuable insights into the experiences of young adults receiving PD. Our findings suggest several key areas in which targeted interventions could improve patient experiences, including better communication, more accessible information, and stronger support systems.^26^^,^^27^

Our results reveal that young adults experience higher rates of PD-to-HD transition compared with children. This trend mirrors outcomes seen in kidney transplantation, suggesting that young adulthood may be a time of increased vulnerability for patients receiving kidney replacement therapy.^28^ However, it is important to note that the absence of an HD comparator group limits our ability to determine whether these transitions are specific to PD or are characteristic of dialysis treatment more generally.

Infection was identified as the most common cause of PD-to-HD transition, accounting for 50% of transitions. This rate is consistent with UK data but higher than previously reported figures (around 20%).^29^^,^^30^ The qualitative findings provided additional context, highlighting how logistical issues—such as limited space, difficult living conditions, and the burden of daily treatment—contributed to the increased risk of infection and the decision to transition to HD.

Qualitative findings highlighted how living with messy housemates added stress to young adults and increased their risk of infection. Young adults should be informed that they can apply for disabled student accommodation when attending university. This will enable them to safely store their PD supplies, as well as give them space to receive PD in a clean environment rather than in a small, cluttered bedroom or messy bathroom.

Health care professionals could also provide additional support when young adults move out of their family home through assisted PD. Our qualitative findings suggest that many young adults struggle when they move out of their family homes, as they lose the support system that previously helped with preparing equipment, cleaning, and performing PD. Young adult support workers could also be beneficial at this stage to provide emotional support and identify any young adults who are not coping, before they become very unwell. Our study underscores the importance of health care teams fostering strong patient-provider relationships, which varied considerably among participants.^31^^,^^32^

Next, noncompliance was also found to be a key reason for PD-to-HD transition. Qualitative findings highlighted how feelings of isolation and missing out on social events led to reduced compliance with their treatment schedule. Many participants felt inadequately informed about their treatment options and some felt they were not warned about the realities of PD before starting. This reflects a broader challenge in ensuring that patients receive clear, accessible, and balanced information about dialysis choices.^23^^,^^26^^,^^28^ Peer support was frequently cited as valuable by participants, suggesting that promoting connections between young adults receiving dialysis could mitigate feelings of isolation and offer practical insights.^33^^,^^34^ Encouraging participation in online support groups, such as those endorsed by Kidney Care UK, could provide young adults with the emotional and practical support they need.^35^

Mental health emerged as a significant concern for many participants, with one-third reporting psychological issues such as depression. Young adults with kidney disease are known to be at increased risk of mental health conditions, including depression and eating disorders, compared with both children and adults.^36^ Depression has been linked to higher rates of dialysis-related complications, including peritonitis and hospitalization, which may partly explain the higher rates of PD-to-HD transition in this age group.^1^^,^^37^ Integrating mental health support into routine care pathways, as well as screening for conditions such as depression and eating disorders, could help identify and address these issues earlier, potentially reducing complications and the need for PD-to-HD transition.^38^

Linked to poor mental health, young adults also expressed a fear of physical activity owing to concerns about damage to the PD catheter. Educating them about resources such as Beam for Kidney Disease, which offers free exercise classes, could help alleviate these concerns and promote better physical health and overall well-being, which may in turn reduce the need for modality changes.^39^

Finally, resilience was a key theme that emerged from the qualitative data. Some demonstrated an impressive ability to adapt to their circumstances despite the challenges of PD. However, the emotional burden of treatment and the impact of illness can reduce resilience, especially in areas such as emotional regulation and problem solving.^40^^,^^41^ Interventions aimed at strengthening resilience could be beneficial, helping patients manage the emotional and psychological challenges of long-term dialysis treatment. Cognitive behavioral approaches, which have shown promise in other chronic illness settings, could be one avenue to explore in future studies as a way to reduce the rate of PD-to-HD transition and improve overall patient outcomes.^42^

One of the strengths of this study was its focus on the experiences of young adults undergoing PD, a group that is often underrepresented in dialysis research.^39^ The combination of a robust statistical approach to identify factors linked with PD-to-HD transitions and the rich, qualitative insights into patients’ lived experiences provides an understanding of the challenges faced by young adults receiving dialysis.

However, the study has several limitations, including the lack of data on specific PD regimens, patient care in adult centers, and proximity to HD units. In addition, only causal variables (*P* < 0.1) were combined in a multivariable Cox regression model, which is now no longer the best practice for model building. In addition, the absence of detailed information on support systems, caregivers, and parental involvement limits our ability to fully explore how these factors influence patient autonomy and treatment choices. Questions on support and caregivers should have been included in the interview topic guide given the age demographic and the strong possibility that many relied heavily on family. Future longitudinal cohort studies could help address these gaps and provide a deeper understanding of the long-term impact of PD on young adults.

In conclusion, kidney replacement therapy is a lifelong journey for young patients, often involving transitions between dialysis modalities. The findings from this study highlight the importance of identifying appropriate treatment options, ensuring adequate preparation for each phase of care, and addressing the long-term psychological and logistical needs of young adults. By improving communication, support systems, and resilience, health care teams can optimize patient experience and help young adults better navigate their treatment journey. Targeted interventions, including educational resources, mental health support, and peer networks, are essential to improving outcomes and reducing the need for PD-to-HD transitions.

## Declaration of Generative AI and AI-Assisted Technologies in the Writing Process

During the preparation of this work the authors used Chat GPT with caution to improve the article’s conciseness to ensure it was not over the word count limit. After using this tool, the authors reviewed and edited the content as needed and takes full responsibility for the content of the publication.

## Supplementary Materials

Supplementary File (PDF) Items S1 and S2.

## References

[1] Hamilton A.J., Caskey F.J., Casula A., Ben-Shlomo Y., Inward C.D. (2019). Psychosocial health and lifestyle behaviors in young adults receiving renal replacement therapy compared to the general population: findings from the SPEAK study. Am J Kidney Dis.

[2] Jung H.Y., Jeon Y., Park Y. (2019). Better quality of life of peritoneal dialysis compared to hemodialysis over a two-year period after dialysis initiation. Sci Rep.

[3] Li P.K., Law M.C., Chow K.M. (2007). Good patient and technique survival in elderly patients on continuous ambulatory peritoneal dialysis. Perit Dial Int.

[4] Nessim S.J., Bargman J.M., Austin P.C., Story K., Jassal S.V. (2009). Impact of age on peritonitis risk in peritoneal dialysis patients: an era effect. Clin J Am Soc Nephrol.

[5] Sun X., McKeaveney C., Shields J. (2024). Rate and reasons for peritoneal dialysis dropout following haemodialysis to peritoneal dialysis switch: a systematic review and meta-analysis. BMC Nephrol.

[6] Chiang P.C., Hou J.J., Jong I.C. (2016). Factors associated with the choice of peritoneal dialysis in patients with end-stage renal disease. Biomed Res Int.

[7] Smart N.A., Titus T.T. (2011). Outcomes of early versus late nephrology referral in chronic kidney disease: a systematic review. Am J Med.

[8] See E.J., Johnson D.W., Hawley C.M. (2018). Risk predictors and causes of technique failure within the first year of peritoneal dialysis: an Australia and New Zealand Dialysis and Transplant Registry (ANZDATA) study. Am J Kidney Dis.

[9] Lim W.H., Dogra G.K., McDonald S.P., Brown F.G., Johnson D.W. (2011). Compared with younger peritoneal dialysis patients, elderly patients have similar peritonitis-free survival and lower risk of technique failure, but higher risk of peritonitis-related mortality. Perit Dial Int.

[10] Shen J.I., Mitani A.A., Saxena A.B., Goldstein B.A., Winkelmayer W.C. (2013). Determinants of peritoneal dialysis technique failure in incident US patients. Perit Dial Int.

[11] Statistics OfN (2024). UNEM01 SA: Unemployment by age and duration (seasonally adjusted). https://www.ons.gov.uk/employmentandlabourmarket/peoplenotinwork/unemployment/datasets/unemploymentbyageanddurationseasonallyadjustedunem01sa.

[12] Hamilton A.J., Caskey F.J., Casula A., Inward C.D., Ben-Shlomo Y. (2018). Associations with wellbeing and medication adherence in young adults receiving kidney replacement therapy. Clin J Am Soc Nephrol.

[13] Tong A., Henning P., Wong G. (2013). Experiences and perspectives of adolescents and young adults with advanced CKD. Am J Kidney Dis.

[14] Lewis H., Arber S. (2015). The role of the body in end-stage kidney disease in young adults: gender, peer and intimate relationships. Chronic Illn.

[15] Hamilton A.J., Clissold R.L., Inward C.D., Caskey F.J., Ben-Shlomo Y. (2017). Sociodemographic, psychologic health, and lifestyle outcomes in young adults on renal replacement therapy. Clin J Am Soc Nephrol.

[16] Noordzij M., Leffondré K., van Stralen K.J., Zoccali C., Dekker F.W., Jager K.J. (2013). When do we need competing risks methods for survival analysis in nephrology?. Nephrol Dial Transplant.

[17] Cleves M. (2000). Analysis of multiple failure-time data with Stata. Stata Tech Bull.

[18] Efron B. (1977). The efficiency of Cox's likelihood function for censored data. J Am Stat Assoc.

[19] Braun V., Clarke V. (2006). Using thematic analysis in psychology. Qual Res Psychol.

[20] Miles M., Huberman A. (1994).

[21] (2020). Ltd. QIP. NVivo (Version 12).

[22] McCrudden M., McTigue E. (2018). Implementing integration in an explanatory sequential mixed methods study of belief bias about climate change with high school students. J Mix Methods Res.

[23] Tong A., Sainsbury P., Craig J. (2007). Consolidated criteria for reporting qualitative research (COREQ): a 32-item checklist for interviews and focus groups. Int J Qual Health Care.

[24] Venkat-Raman G., Tomson C.R., Gao Y. (2012). New primary renal diagnosis codes for the ERA-EDTA. Nephrol Dial Transplant.

[25] Abel G.A., Barclay M.E., Payne R.A. (2016). Adjusted indices of multiple deprivation to enable comparisons within and between constituent countries of the UK including an illustration using mortality rates. BMJ Open.

[26] Fadem S.Z., Walker D.R., Abbott G. (2011). Satisfaction with renal replacement therapy and education: the American Association of Kidney Patients survey. Clin J Am Soc Nephrol.

[27] Curtin R.B., Johnson H.K., Schatell D. (2004). The peritoneal dialysis experience: insights from long-term patients. Nephrol Nurs J.

[28] Morton R.L., Tong A., Howard K., Snelling P., Webster A.C. (2010). The views of patients and carers in treatment decision making for chronic kidney disease: systematic review and thematic synthesis of qualitative studies. BMJ.

[29] Lambie M., Zhao J., McCullough K. (2022). Variation in peritoneal dialysis time on therapy by country: results from the Peritoneal Dialysis Outcomes and Practice Patterns Study. Clin J Am Soc Nephrol.

[30] Mehrotra R., Devuyst O., Davies S.J., Johnson D.W. (2016). The current state of peritoneal dialysis. J Am Soc Nephrol.

[31] Sadala M.L., Miranda M.G., Lorençon M., de Campos Pereira E.P. (2010). Nurse-patient communication while performing home dialysis: the patients' perceptions. J Ren Care.

[32] Ma X., Shi Y., Tao M. (2020). Analysis of risk factors and outcome in peritoneal dialysis patients with early-onset peritonitis: a multicentre, retrospective cohort study. BMJ Open.

[33] Elliott M.J., Love S., Fox D.E. (2022). It’s the empathy’-defining a role for peer support among people living with chronic kidney disease: a qualitative study. BMJ Open.

[34] Chan C.T., Blankestijn P.J., Dember L.M. (2019). Dialysis initiation, modality choice, access, and prescription: conclusions from a Kidney Disease: Improving Global Outcomes (KDIGO) Controversies Conference. Kidney Int.

[35] Facebook Young Adult Kidney Group. https://www.facebook.com/groups/youngadultkidneygroup/.

[36] Collaborators G.M.D. (2022). Global, regional, and national burden of 12 mental disorders in 204 countries and territories, 1990-2019: a systematic analysis for the Global Burden of Disease Study 2019. Lancet Psychiatry.

[37] King-Wing Ma T., Kam-Tao Li P. (2016). Depression in dialysis patients. Nephrology (Carlton).

[38] Osório E., Milheiro I., Brandão I., Roma Torres A. (2013). Anorexia nervosa and dialysis: we have no time when the body is so damaged. BMJ Case Rep.

[39] Mayes J., Billany R.E., Vadaszy N. (2022). The rapid development of a novel kidney-specific digital intervention for self-management of physical activity and emotional well-being during the COVID-19 pandemic and beyond: kidney beam. Clin Kidney J.

[40] Tong A., Lesmana B., Johnson D.W., Wong G., Campbell D., Craig J.C. (2013). The perspectives of adults living with peritoneal dialysis: thematic synthesis of qualitative studies. Am J Kidney Dis.

[41] Gooding P.A., Hurst A., Johnson J., Tarrier N. (2012). Psychological resilience in young and older adults. Int J Geriatr Psychiatry.

[42] Rosenberg A.R., Bradford M.C., McCauley E. (2018). Promoting resilience in adolescents and young adults with cancer: results from the PRISM randomized controlled trial. Cancer.

