# Supplementary material for: Peritoneal Dialysis in Young Adults: A Mixed-Methods Study
Source: Kidney Med. 2025 Feb 15;7(4):100983. doi: 10.1016/j.xkme.2025.100983 (PMC11978335; doi:10.1016/j.xkme.2025.100983)
Supplement: Supplementary File (PDF) — Items S1 and S2. [file mmc1.docx]

Item S1: Theoretical framework of psychosocial associations with PD failure used to inform statistical analysis

| Clinical Data   - Date first seen - Treatment modality changes - Biochemistry markers - Primary renal disease defined by the European Renal Association– European Dialysis and Transplant Association coding   system(13) | Social markers   - Age left school - Higher education - Marital status - Religion - Index of Multiple Deprivation(12) | Biological markers   - Sex - Age - Ethnic group | Psychological   - Body image - Stigma, social support, patient activation, locus of control, personality, medication adherence (this was omitted due to legal reasons), activity of daily living) |
| --- | --- | --- | --- |

| Characteristics of individual |
| --- |
| Peritoneal dialysis failure |

Item S2: Qualitative interview topic guide

Introduction

- Welcome participant, introduce researcher, reassure confidentiality
- Inform on purpose of study:

- We are interested in young peoples’ experiences of dialysis and exploring why people sometimes change from peritoneal dialysis to haemodialysis.

Initial thoughts on peritoneal dialysis

- Open question: please could you describe what your experience was of having peritoneal dialysis?

Beginning… what do you remember about…

- Information provided on dialysis
- Catheter insertion
- Training
- First exchange in hospital and at home Maintaining PD
- How was your confidence in managing your PD?
- How did having PD fit in with your life at the time?
  - Frequency
  - Education, employment
  - Relationships, body image and intimacy
  - Social life
  - Mental health
- How was your relationship with the kidney unit?
  - Doctors
  - PD nurses
  - Youth worker
  - GP Stopping PD
- Please can you explain what happened when you switched to HD?
- What was it like having HD?

Thoughts now

- If someone approached you who needed to have dialysis and was seeking advice on the different options, what would you say to them?
- If you needed to, would you consider having PD again?

- Why?

Concluding

- Is there anything else you would like to add about your experience of PD?
